# Predictors of COVID-19 severity: a systematic review and meta-analysis

**DOI:** 10.12688/f1000research.26186.2

**Published:** 2021-01-06

**Authors:** Mudatsir Mudatsir, Jonny Karunia Fajar, Laksmi Wulandari, Gatot Soegiarto, Muhammad Ilmawan, Yeni Purnamasari, Bagus Aulia Mahdi, Galih Dwi Jayanto, Suhendra Suhendra, Yennie Ayu Setianingsih, Romi Hamdani, Daniel Alexander Suseno, Kartika Agustina, Hamdan Yuwafi Naim, Muchamad Muchlas, Hamid Hunaif Dhofi Alluza, Nikma Alfi Rosida, Mayasari Mayasari, Mustofa Mustofa, Adam Hartono, Richi Aditya, Firman Prastiwi, Fransiskus Xaverius Meku, Monika Sitio, Abdullah Azmy, Anita Surya Santoso, Radhitio Adi Nugroho, Camoya Gersom, Ali A. Rabaan, Sri Masyeni, Firzan Nainu, Abram L. Wagner, Kuldeep Dhama, Harapan Harapan

**Affiliations:** 1 Department of Microbiology, School of Medicine, Universitas Syiah Kuala, Banda Aceh, Aceh, 23111, Indonesia; 2 Brawijaya Internal Medicine Research Center, Department of Internal Medicine, Faculty of Medicine, Universitas Brawijaya, Malang, East Java, 65145, Indonesia; 3 Department of Pulmonology and Respiratory Medicine, Faculty of Medicine, Universitas Airlangga, Surabaya, East Java, 60286, Indonesia; 4 Department of Internal Medicine, Faculty of Medicine, Universitas Airlangga, Surabaya, Easy Java, 60286, Indonesia; 5 Faculty of Medicine, Universitas Brawijaya, Malang, East Java, 65145, Indonesia; 6 Department of Urology, Faculty of Medicine, Universitas Airlangga, Surabaya, East Java, 60285, Indonesia; 7 Department of Orthopedic Surgery, Faculty of Medicine, Universitas Brawijaya, Malang, East Java, 65145, Indonesia; 8 Department of Obstetry and Gynecology, Faculty of Medicine, Universitas Brawijaya, Malang, East Java, 65145, Indonesia; 9 Department of Neurology, Faculty of Medicine, Universitas Brawijaya, Malang, East Java, 65145, Indonesia; 10 Department of Urology, Faculty of Medicine, Universitas Brawijaya, Malang, East Java, 65145, Indonesia; 11 Faculty of Animal Science, Universitas Brawijaya, Malang, East Java, 65145, Indonesia; 12 Faculty of Medicine, Universitas Negeri Sebelas Maret, Surakarta, Surakarta, 57126, Indonesia; 13 Department of Cardiology and Vascular Medicine, Faculty of Medicine, Universitas Brawijaya, Malang, East Java, 65145, Indonesia; 14 Molecular Diagnostic Laboratory, Johns Hopkins Aramco Healthcare, Dhahran, Dhahran, 31311, Saudi Arabia; 15 Department of Internal Medicine, Faculty of Medicine and Health Science, Universitas Warmadewa, Denpasar, Bali, 80235, Indonesia; 16 Faculty of Pharmacy, Hasanuddin University, Makassar, Makassar, 90245, Indonesia; 17 Department of Epidemiology, University of Michigan, Ann Arbor, MI, 48109, USA; 18 Division of Pathology, Indian Veterinary Research Institute, Izatnagar, Uttar Pradesh, 243 122, India; 19 Medical Research Unit, School of Medicine, Universitas Syiah Kuala, Banda Aceh, Aceh, 23111, Indonesia

**Keywords:** SARS-CoV-2, COVID-19, prognosis, severity, clinical outcome

## Abstract

**Background**: The unpredictability of the progression of coronavirus disease 2019 (COVID-19) may be attributed to the low precision of the tools used to predict the prognosis of this disease.

**Objective**: To identify the predictors associated with poor clinical outcomes in patients with COVID-19.

**Methods**: Relevant articles from PubMed, Embase, Cochrane, and Web of Science were searched as of April 5, 2020. The quality of the included papers was appraised using the Newcastle-Ottawa scale (NOS). Data of interest were collected and evaluated for their compatibility for the meta-analysis. Cumulative calculations to determine the correlation and effect estimates were performed using the Z test.

**Results**: In total, 19 papers recording 1,934 mild and 1,644 severe cases of COVID-19 were included. Based on the initial evaluation, 62 potential risk factors were identified for the meta-analysis. Several comorbidities, including chronic respiratory disease, cardiovascular disease, diabetes mellitus, and hypertension were observed more frequent among patients with severe COVID-19 than with the mild ones. Compared to the mild form, severe COVID-19 was associated with symptoms such as dyspnea, anorexia, fatigue, increased respiratory rate, and high systolic blood pressure. Lower levels of lymphocytes and hemoglobin; elevated levels of leukocytes, aspartate aminotransferase, alanine aminotransferase, blood creatinine, blood urea nitrogen, high-sensitivity troponin, creatine kinase, high-sensitivity C-reactive protein, interleukin 6, D-dimer, ferritin, lactate dehydrogenase, and procalcitonin; and a high erythrocyte sedimentation rate were also associated with severe COVID-19.

**Conclusion**: More than 30 risk factors are associated with a higher risk of severe COVID-19. These may serve as useful baseline parameters in the development of prediction tools for COVID-19 prognosis.

## Introduction

The coronavirus disease 2019 (COVID-19) pandemic, caused by severe acute respiratory syndrome coronavirus 2 (SARS-CoV-2), is a global crisis across health, economic, and educational dimensions ^1, 2^. The disease has spread rapidly, can cause severe illness, and is characterized by a high mortality rate in certain groups. Mortality is particularly high in the absence of proven effective standard management measures ^3^. One of the problems with the management of this disease is the absence of standardized methods for diagnosis and the inability to estimate prognosis based on clinical features. Certain reports have shown that poor prognostic prediction has correlated with high mortality among patients with COVID-19 ^4, 5^. Among patients with similar clinical characteristics and with similar treatment regiments, there may be a diversity in clinical outcomes ^6^. Therefore, the development and use of an accurate predictor for COVID-19 prognosis will be beneficial for the clinical management of patients with COVID-19, and will help reduce the mortality rate. Successful implementation of such a prediction mechanism could have a large public health impact. Better understanding of clinical progression could also improve public health messaging, particularly as many individuals may consider COVID-19 to not be severe.

Prognostic tools for the prediction of COVID-19 severity in patients have been in development since January 2020. At least nine studies proposed the use of prognostic tools for the prediction of COVID-19 severity ^7– 15^. However, a recent systematic review and critical appraisal study evaluated the accuracy of these tools using prediction model risk of bias assessment tool (PROBAST) and reported a high risk of bias ^16^. The establishment of a prediction model for the estimation of disease prognosis may help health workers segregate patients according to prediction status. However, the high risk of bias in these prediction tools might lead to inaccurate prediction of COVID-19 severity. A comprehensive study of the identification of risk factors that might play a significant role in determining the severity of patients with COVID-19 is necessary. We performed a systematic review and meta-analysis to assess the risk factors associated with poor clinical outcomes among patients with COVID-19. To the best of our knowledge, this is the first meta-analysis to assess the comprehensive risk factors that might affect the severity of COVID-19 in patients. The results of our study might serve as preliminary data for the compilation or improvement of the scoring system in the prediction of COVID-19 severity.

## Methods

### Study design

We performed a systematic review and meta-analysis to evaluate potential risk factors that might influence the severity of COVID-19. These risk factors include comorbidities, clinical manifestations, and laboratory findings. Accordingly, we searched the relevant studies from major scientific websites and databases to collect the data of interest, and determined the association and effect estimates by calculating the combined odds ratio (OR) and 95% confidence intervals (95% CI). The protocols for the systematic review and meta-analysis were similar to those used in previous studies ^17– 23^, as well as to those recommended by Preferred Reporting Items for Systematic Review and Meta-Analysis (PRISMA) ^24^.

### Eligibility criteria

Studies were included in this review if they met the following inclusion criteria: (1) assessed the clinical manifestations and laboratory findings of patients with mild to severe COVID-19; (2) provided adequate data for the calculation of OR and 95% CI (event per sample size or mean ± SD in both case and control groups), (3) written inEnglish. Review articles, articles with non-standard data presentation, and duplicate publications were excluded.

### Search strategy and data extraction

Major scientific databases (PubMed, Embase, Cochrane, and Web of Science) were searched for articles as of April 5, 2020. Moreover, we also searched in google scholar for the additional database. A comprehensive initial search was performed to identify the potential predictors, and a final search was performed to identify the relevant papers that could be included in the meta-analysis. We used the keywords adapted from medical subject headings: ["COVID-19" or "Coronavirus disease-19" or "SARS-CoV-2"] and ["mild" or "severe" or "prognosis" or "clinical outcome"] and ["clinical manifestation" or "morbidity" or "laboratory findings"]. Only studies written in English were included. If a duplicate publication was found, the article with the larger sample size was included. We also searched for relevant studies from the reference lists in the articles. During data extraction, the following information of interest was extracted: (1) first author name; (2) publication year; (3) country of origin, (4) sample size of mild and severe cases, (5) clinical manifestations, (6) morbidities, and (7) laboratory findings. Data extraction was performed by two independent investigators (JKF and MI) using a pilot form. If the disagreement was found, we performed a discussion to resolve the disagreement.

### Assessment of the methodological quality

Before inclusion in the meta-analysis, the methodological quality of the articles was assessed using the New Castle-Ottawa scale (NOS). NOS scores range from 0 to 9 and consider three items: selection of patients (4 points), comparability of the groups (2 points), and ascertainment of exposure (3 points). Each study was interpreted to be of low quality (for scores ≤ 4), moderate quality (for scores between 5–6), or high quality (for scores ≥ 7) ^25^. Articles with moderate to high quality were included in the analysis. The study assessment was conducted by two independent investigators (MI and YP) using a pilot form. The discrepancies between the findings of the two investigators were solved by consulting with another investigator (JKF).

### Study measures

The outcome measure of the study was the severity of COVID-19 (mild *vs*. severe). The risk factors or predictors included three major groups: comorbidities, clinical manifestations, and laboratory parameters. Comorbid factors such as chronic kidney disease, chronic liver disease, chronic respiratory disease, cerebrovascular accident, cardiovascular disease, diabetes mellitus, hypertension, and malignancy were compatible with the analysis. For clinical manifestations, fever, cough, dry cough, expectoration, sore throat, dyspnea, diarrhea, myalgia, nasal congestion, anorexia, abdominal pain, fatigue, dizziness, headache, fever, heart rate, respiratory rate, systolic blood pressure, and diastolic blood pressure were included in this study. Among laboratory characteristics, the presence of leukocytosis, leukocytopenia, anemia, lymphocytopenia; the levels or the counts of white blood cell (WBC), hemoglobin, neutrophil, lymphocyte, monocyte, platelet, activated partial thromboplastin time (aPTT), partial thromboplastin time (PTT), aspartate aminotransferase (AST), alanine aminotransferase (ALT), total bilirubin, albumin, serum creatinine, blood urea nitrogen (BUN), high-sensitivity (Hs)-troponin I, creatine kinase, high-sensitivity C-reactive protein (Hs-CRP), C-reactive protein (CRP) >8 mg/L, interleukin 6 (IL-6), glucose, D-dimer, serum ferritin, sodium, potassium, lactate dehydrogenase, and procalcitonin, CD4 and CD8; erythrocyte sedimentation rate (ESR); elevated IL-16; and elevated ESR were all included.

### Statistical analysis

The significant risk factors that might govern the severity of COVID-19 were determined by the calculation of a pooled OR and 95% CI. The significance of the pooled ORs was determined using the Z test (p<0.05 was considered statistically significant). Prior to identification of the significant risk factors, data were evaluated for heterogeneity and potential publication bias. The heterogeneity among included studies was evaluated using the Q test. If heterogeneity existed (p<0.10), a random effect model was adopted; if not, a fixed effect model was adopted. Egger’s test and a funnel plot were used to assess the reporting or publication bias (p<0.05 was considered statistically significant). Furthermore, we performed a moderator analysis to identify the independent predictors of poor clinical outcomes among patients with COVID-19. The data were analyzed using Review Manager version 5.3 (Revman Cochrane, London, UK). To prevent analytical errors, statistical analysis was performed by two authors (JKF and MI). The cumulative calculation was presented in a forest plot.

## Results

### Eligible studies

**Figure 1.**
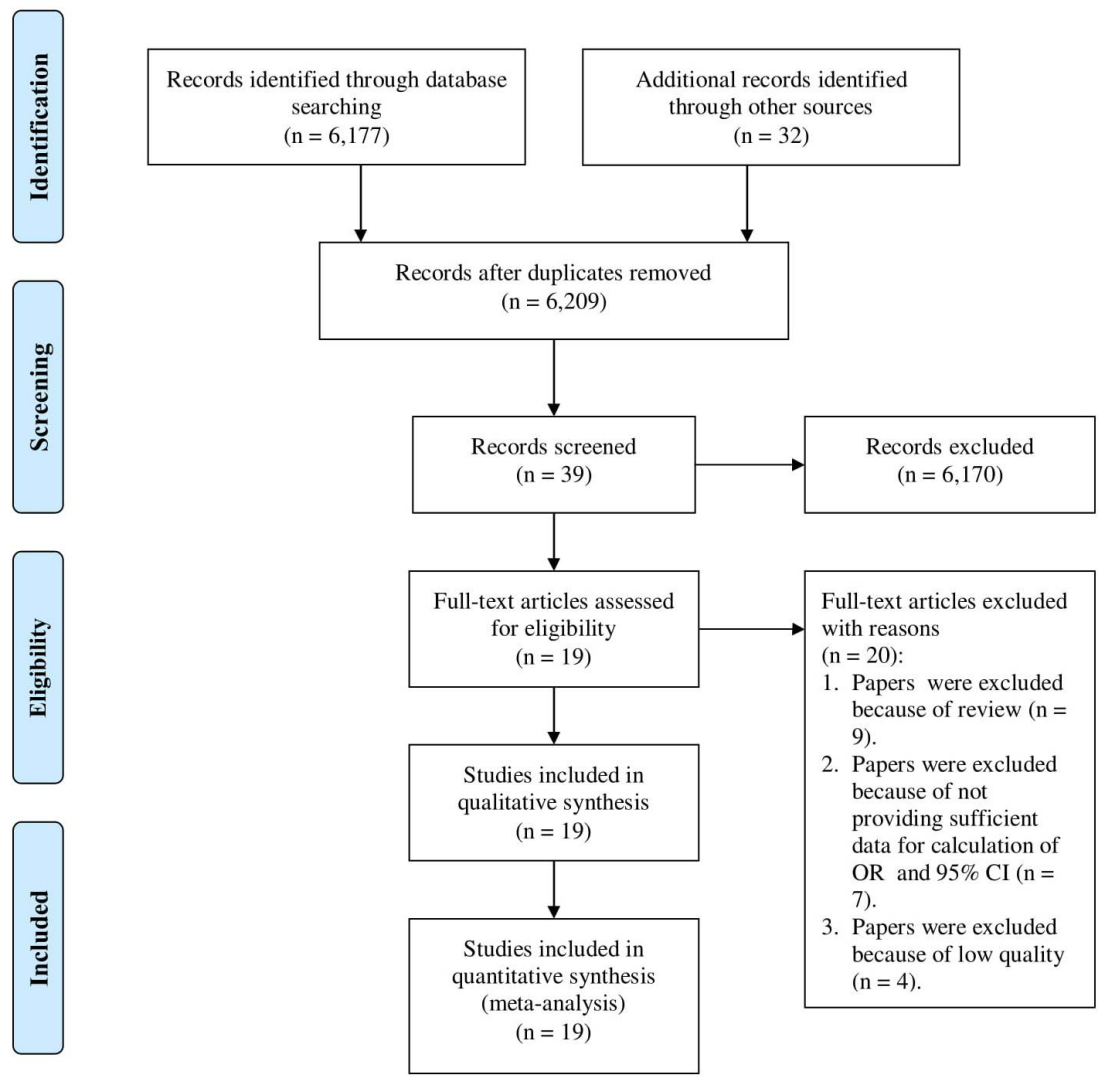
A flowchart of paper selection in our study.

Our searches yielded 6,209 potentially relevant studies, of which 6,170 studies were excluded after assessment of the titles and abstracts. Subsequently, further review of the complete texts was performed for 39 potential studies. In the full text review, we excluded 20 studies because they were reviews articles (n = 9), inadequacy of data for the calculation of OR and 95% CI (n = 7), and poor quality (n = 4). Eventually, 19 papers were included in our meta-analysis ^26– 42^ The paper selection process adopted in our study is summarized in Figure 1, and the characteristics of studies included in our analysis are outlined in Table 1.

**Table 1.** Baseline characteristics of studies included in our analysis.

Author & year	Country	City	Hospital	Sample size		Outcome measure	NOS
				*Severe*	*Mild*		
Bai *et al.* 2020 ^26^	China	Wuhan	Jinyintan Hospital	91	36	Died vs. cured	7
Cai *et al.* 2020 ^27^	China	Shenzen	Third people's Hospital	58	240	Severe vs. non severe	9
Chen *et al.* 2020 ^28^	China	Wuhan	Tongji hospital	11	10	Severe vs. moderate	9
Chen *et al.* 2020 ^29^	China	Mixed	Multicenter	50	241	Severe vs. mild-moderate	9
Chen *et al.* 2020 ^30^	China	Wuhan	Zhongnan Hospital	14	11	Viral clearance vs. without viral clearance	9
Duan *et al.* 2020 ^31^	China	Wuhan	Wuhan Pulmonary Hospital	44	72	Uncured vs. cured	9
Gao *et al.* 2020 ^32^	China	Fuyang	Second People's Hospital	15	28	Severe vs. mild	7
Guan *et al.* 2020 ^33^	China	Guangdong	National Health Commision of China	926	173	Severe vs. non-severe	7
Huang *et al.* 2020 ^34^	China	Wuhan	Jinyintan hospital	13	28	ICU vs. non-ICU	9
Jian-Ya *et al.* 2020 ^35^	China	Chongqing	Three Gorges Hospital	7	44	Severe vs. non severe	9
Liu *et al.* 2020 ^36^	China	Wuhan	Union Hospital	69	69	Severe vs. non severe	7
Shi *et al.* 2020 ^37^	China	Wuhan	Renmin Hospital	48	53	Died <3 d vs. >3 d	9
Wang *et al.* 2020 ^38^	China	Mixed	Multicenter	50	115	CT imaging score >11 vs. <11	8
Wang *et al.* 2020 ^39^	China	Wuhan	Wuhan First People's Hospital	22	283	Survivor vs. non-survivor	8
Wang *et al.* 2020 ^43^	China	Wuhan	Zhongnan Hospital	36	102	ICU vs. non-ICU	9
Xu *et al.* 2020 ^40^	China	Mixed	Multicenter	25	44	Severe vs. mild	8
Zhang *et al.* 2020 ^41^	China	Wuhan	Zhongnan Hospital	55	166	Severe vs. non-severe	9
Zhang *et al.* 2020 ^44^	China	Wuhan	Wuhan Seventh Hospital	56	82	Severe vs. non-severe	7
Zhou *et al.* 2020 ^42^	China	Wuhan	Wuhan Pulmonary Hospital	54	137	Survivor vs. non-survivor	8

Note: ICU, intensive care unit; CT, computed tomography; NOS, Newcastle Ottawa Scale.

### Risk factors of severe COVID-19

**Figure 2.**
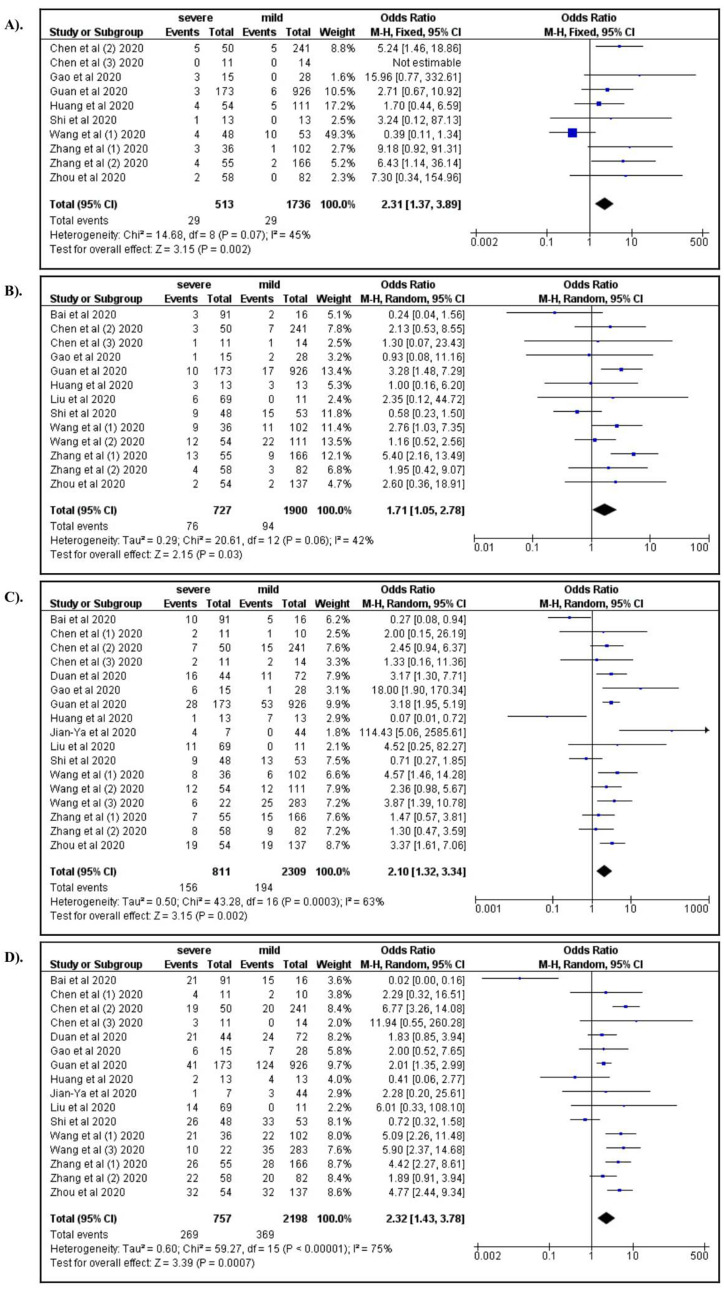
A forest plot of the association between comorbid factors and the risk of severe COVID-19. **A**) Chronic respiratory disease; **B**) Cardiovascular diease; **C**) Diabetes mellitus; **D**) Hypertension.

We found that eight comorbidities, 19 clinical manifestations, and 35 laboratory parameters were available for the meta-analysis ( Table 2 and Table 3). Among the comorbid factors, chronic respiratory disease (OR: 2.48; 95% CI: 1.44, 4.27), cardiovascular disease (OR: 1.70; 95% CI: 1.05, 2.78), diabetes mellitus (OR: 2.10; 95% CI: 1.33, 3.34), and hypertension (OR: 2.33; 95% CI: 1.42, 3.81) were associated with a greater risk of severe COVID-19 ( Figure 2A–D).

**Table 2.** Clinical characteristics of Covid-19 patients and the risk of severity.

Clinical characteristics	NS	Model	Value		pE	pHet	p	OR	95%CI
			*Severe*	*Mild*					
Comorbids									
Chronic kidney disease	6	Random	14 [3.94]	15 [1.68]	1.3430	0.0280	0.1910	2.56	0.63-10.45
Chronic liver disease	6	Fixed	16 [4.82]	26 [4.04]	<0.0001	0.3220	0.3220	1.45	0.70-3.01
Chronic respiratory disease	10	Fixed	31 [5.47]	31 [1.66]	0.7060	0.1020	0.0010	2.48	1.44-4.27
Cerebrovascular accident	5	Random	20 [5.54]	30 [2.09]	0.9110	0.0380	0.1850	2.02	0.71-5.70
Cardiovascular disease	13	Random	76 [10.45]	94 [4.95]	0.5400	0.0580	0.0310	1.70	1.05-2.78
Diabetes mellitus	17	Random	156 [19.24]	194 [8.40]	0.7040	<0.0001	0.0020	2.10	1.33-3.34
Hypertension	15	Random	269 [35.54]	369 [16.79]	0.7680	<0.0001	0.0010	2.33	1.42-3.81
Malignancy	11	Fixed	29 [4.43]	40 [2.23]	0.6150	0.1430	0.5330	1.18	0.70-1.99
Symptoms									
Fever	16	Random	599 [79.34]	1932 [80.84]	0.9220	<0.0001	0.1730	1.51	0.83-2.74
Cough	12	Random	377 [64.33]	1120 [ 54.05]	0.9560	<0.0001	0.1890	1.53	0.81-2.90
Dry cough	4	Fixed	75 [44.38]	178 [55.97]	0.3130	0.1880	0.0360	0.66	0.44-0.97
Expectoration	10	Fixed	136 [26.67]	438 [29.05]	<0.0001	0.8370	0.4970	1.09	0.85-1.39
Sore throat	10	Random	59 [10.57]	196 [10.96]	0.7860	0.0040	0.6350	1.18	0.59-2.37
Dyspnea	13	Random	286 [42.56]	318 [16.51]	0.6340	<0.0001	<0.0001	3.28	2.09-5.15
Diarrhea	13	Random	65 [9.62]	134 [6.68]	0.5180	0.0690	0.8030	1.07	0.67-1.69
Myalgia	11	Fixed	105 [17.89]	283 [15.70]	<0.0001	0.7330	0.5160	1.10	0.831-1.44
Nasal congestion	4	Fixed	15 [5.02]	53 [4.34]	0.9350	0.1000	0.7590	1.12	0.55-2.29
Anorexia	9	Random	103 [25.37]	143 [15.10]	0.6960	0.0040	0.0490	1.83	1.00-3.34
Abdominal pain	5	Fixed	15 [6.07]	6 [0.95]	<0.0001	0.5650	0.0040	3.91	1.53-10.02
Fatigue	13	Random	310 [46.48]	694 [34.49]	0.6790	<0.0001	0.0040	2.00	1.25-3.20
Dizziness	4	Fixed	13 [10.08]	24 [5.02]	0.6510	0.1950	0.0180	2.67	1.18-6.01
Headache	11	Fixed	56 [10.45]	197 [11.58]	0.5070	0.1110	0.9950	1.00	0.71-1.41
Signs									
Temperature >38°C	5	Random	200 [57.97]	738 [50.14]	0.6090	0.0020	0.2660	1.44	0.76-2.73
Heart rate (x/min)	4	Fixed	269 ± 35.54	87.88 ± 13.30	<0.0001	0.4070	0.0010	1.79	1.25-2.56
Respiratory rate (x/min)	5	Random	22.6 ± 4.80	20.36 ± 2.00	0.8080	<0.0001	0.0100	2.85	1.28-6.33
SBP (mmHg)	5	Fixed	132.57 ± 23.16	123.88 ± 14.37	0.3340	0.1560	<0.0001	1.84	1.31-2.60
DBP (mmHg)	3	Random	76.50 ± 10.61	75.59 ± 9.89	0.5350	0.0260	0.7190	1.14	0.56-2.32

Note, Value, data were presented in number [%] or mean ± SD; NS, number of studies; pE, p Egger; pHet, p heterogeneity; OR, odd ratio; CI, confidence interval; SBP, systolic blood pressure; DBP, diastolic blood pressure.

**Table 3.** Laboratory findings and the risk of severity in Covid-19 patients.

Clinical characteristics	NS	Model	Value		pE	pHet	p	OR	95%CI
			*Severe*	*Mild*					
Complete Blood Count									
WBC (10^9/L)	14	Random	7.32 ± 3.84	5.17 ± 2.04	1.4980	<0.0001	<0.0001	4.92	2.12-11.31
Leukocytosis	6	Fixed	62 [26.00]	40[6.03]	0.0000	0.5940	<0.0001	5.38	3.36-8.62
Leukopenia	6	Fixed	44 [18.00]	206 [31.07]	0.2890	0.2480	0.0160	0.59	0.41-0.87
Neutrophil count (10^9/L)	12	Random	5.96 ± 3.62	3.84 ± 2.12	1.6380	<0.0001	0.0010	5.45	2.04-14.54
Lymphocyte count (10^9/L)	15	Random	0.74 ± 0.36	1.03 ± 0.44	0.6440	<0.0001	<0.0001	0.34	0.23-0.50
Lymphocytopenia	6	Random	158 [59.00]	40 [6.03]	0.8270	<0.0001	<0.0001	3.19	1.14-7.07
Monocyte count (10^9/L)	6	Random	0.38 ± 0.17	0.36 ± 0.15	0.5860	0.0100	0.5100	1.22	0.68-2.20
Hemoglobin (g/L)	9	Fixed	129.11 ± 16.98	132.02 ± 17.50	0.0900	0.4000	0.0460	0.76	0.58-1.00
Anaemia	2	Random	18 [17.00]	39 [10.32]	0.7640	0.0660	0.4730	1.58	0.45-5.56
Platelet count (10^9/L)	12	Random	172.58 ± 69.19	183.21 ± 62.50	0.5550	0.0010	0.8200	0.82	0.55-1.23
Physiological function									
AST (U/L)	11	Random	56.20 ± 35.83	28.67 ± 11.18	0.6930	<0.0001	<0.0001	4.91	2.96-8.12
ALT (U/L)	12	Random	38.65 ± 22.90	25.60 ± 14.71	0.8060	<0.0001	<0.0001	3.23	1.90-5.52
Total bilirubin (μmol/L)	7	Random	15.80 ± 9.50	13.46 ± 4.62	1.6600	<0.0001	0.5800	1.46	0.41-5.21
Albumin (g/L)	6	Random	32.39 ± 3.64	35.53 ± 3.71	2.3900	<0.0001	0.0950	0.19	0.03-1.34
aPTT (s)	7	Random	31.23 ± 5.02	33.13 ± 3.66	1.1900	<0.0001	0.3420	0.58	0.19-1.79
PTT (s)	11	Random	13.45 ± 1.86	12.53 ± 1.31	0.7700	<0.0001	0.2430	0.56	0.21-1.48
Serum creatinine (μmol/L)	13	Random	82.04 ± 31.69	70.25 ± 20.87	0.6670	<0.0001	0.0010	2.14	1.37-3.33
BUN (mmol/L)	10	Random	6.71 ± 2.70	4.74 ± 1.38	1.0220	<0.0001	<0.0001	6.15	3.05-12.37
Hs-Troponin I (pg/ml)	6	Random	31.9 ± 61.55	3.55 ± 3.71	1.1290	<0.0001	<0.0001	9.25	3.51-24.37
Creatine kinase (U/L)	10	Random	121.13 ± 115.63	77.47 ± 56.26	0.4860	0.0030	<0.0001	2.44	1.65-3.62
Inflammation markers									
Hs-CRP (mg/L)	10	Random	73.25 ± 49.97	29.96 ± 24.40	1.5600	<0.0001	<0.0001	14.27	5.13-39.71
CRP >8 mg/L	3	Random	147 [83.10]	254 [52]	1.1590	0.0050	0.0060	8.34	1.85-37.62
ESR (mm/h)	4	Random	50.60 ± 27.25	29.19 ± 26.52	0.4200	0.0710	<0.0001	4.45	2.56-7.76
Elevated ESR	2	Fixed	73 [68.00]	214 [44.49]	<0.0001	0.8060	<0.0001	2.80	1.78-4.39
IL-6 (pg/ml)	8	Random	30.45 ± 31.29	11.06 ± 10.89	0.9120	<0.0001	<0.0001	6.68	3.20-13.94
Elevated IL-6	2	Fixed	44 [66]	115 [46.56]	<0.0001	0.7160	0.0200	1.98	1.12-3.52
CD4 count(10^9/L)	3	Random	217.19 ± 118.56	337.87 ± 149.93	1.5920	0.0010	0.2760	0.34	0.05-2.39
CD8 count (10^9/L)	3	Random	178.80 ± 95.77	224.17 ± 76.36	1.4260	0.0030	0.1420	0.26	0.04-1.57
Others									
Glucose (mmol/L)	3	Random	7.04 ± 1.83	6.45 ± 1.33	0.9480	0.0030	0.3340	1.80	0.55-5.90
D-dimer (pg/mL)	15	Random	111.34 ± 145.12	38.88 ± 28.93	0.6070	<0.0001	<0.0001	6.19	4.22 - 9.08
Serum Ferritin (μg/L)	4	Fixed	1062.90 ± 868.19	600.67 ± 758.61	0.4310	0.1070	0.0310	1.96	1.06-3.62
Sodium (mmol/L)	3	Random	137.40 ± 3.13	92.39 ± 1.77	3.2770	<0.0001	0.2840	11.93	0.13-1109.37
Potassium (mmol/L)	3	Random	4.12 ± 0.61	4.00 ± 0.54	0.9630	0.0010	0.7470	1.21	0.32-0.75
Lactate dehydrogenase (U/L)	9	Random	381.85 ± 159.44	283. 03 ± 89.40	0.6840	<0.0001	<0.0001	8.28	4.75-14.46
Procalcitonin (ng/mL)	10	Random	0.40 ± 0.29	0.12 ± 0.07	0.9880	<0.0001	<0.0001	6.62	3.32-13.21

Note: Value, data were presented in number [%] or mean ± SD; NS, number of studies; pE, p Egger; pHet, p heterogeneity; OR, odd ratio; CI, confidence interval; CBC, complete blood count; WBC, white blood cells; AST, aspartate transaminase; ALT, alanine transaminase; aPTT, activated partial thromboplastin time; PTT, partial thromboplastin time; BUN, blood urea nitrogen; Hs-CRP, high sensitivity C reactive protein; ESR, erythrocyte sedimentation rate; IL, interleukin.

**Figure 3.**
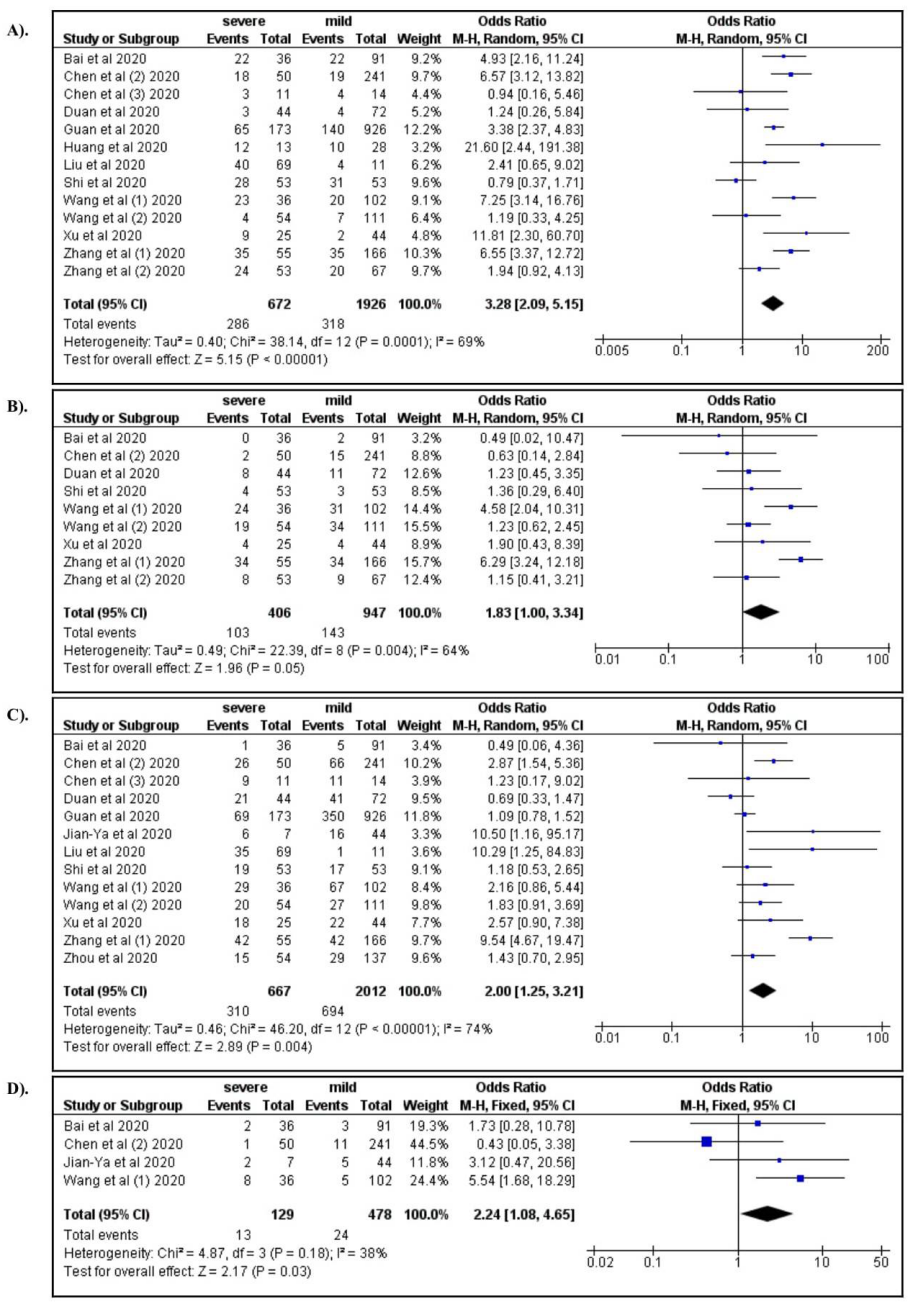
A forest plot of the association between clinical manifestations and the risk of severe COVID-19. **A**) Dyspnea; **B**) Anorexia; **C**) Fatique; **D**) Dizziness.

**Figure 4.**
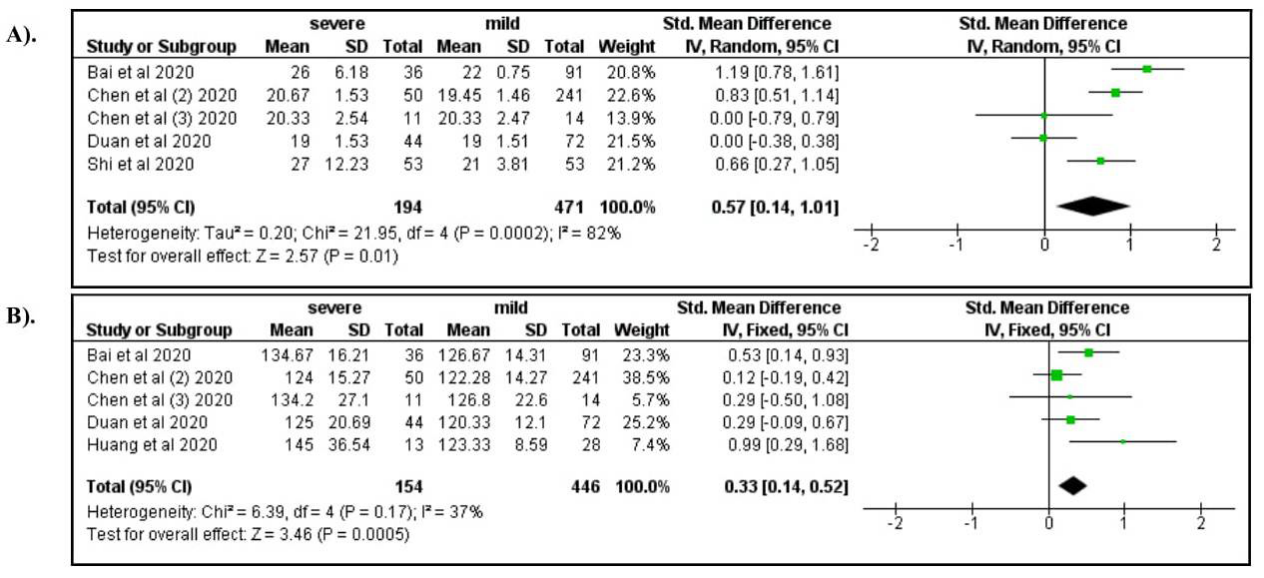
A forest plot of the association between clinical manifestation and the risk of severe COVID-19. **A**) Respiratory rate; **B**) Systolic blood pressure.

Among the clinical manifestations, dyspnea (OR: 3.28; 95% CI: 2.09, 5.15), anorexia (OR: 1.83; 95% CI: 1.00, 3.34), fatigue (OR: 2.00; 95% CI: 1.25, 3.20), and dizziness (OR: 2.67; 95% CI: 1.18, 6.01) were associated with severe COVID-19 ( Figure 3A–D). In addition, increased respiratory rate (OR: 2.85; 95% CI: 1.28, 6.33) and increased systolic blood pressure (OR: 1.84; 95% CI: 1.31, 2.60) were also associated with severe COVID-19 ( Figure 4A and B). Compared to productive cough, dry cough was associated with a lower risk of severe COVID-19 (OR: 0.66; 95% CI: 0.44, 0.97).

**Figure 5.**
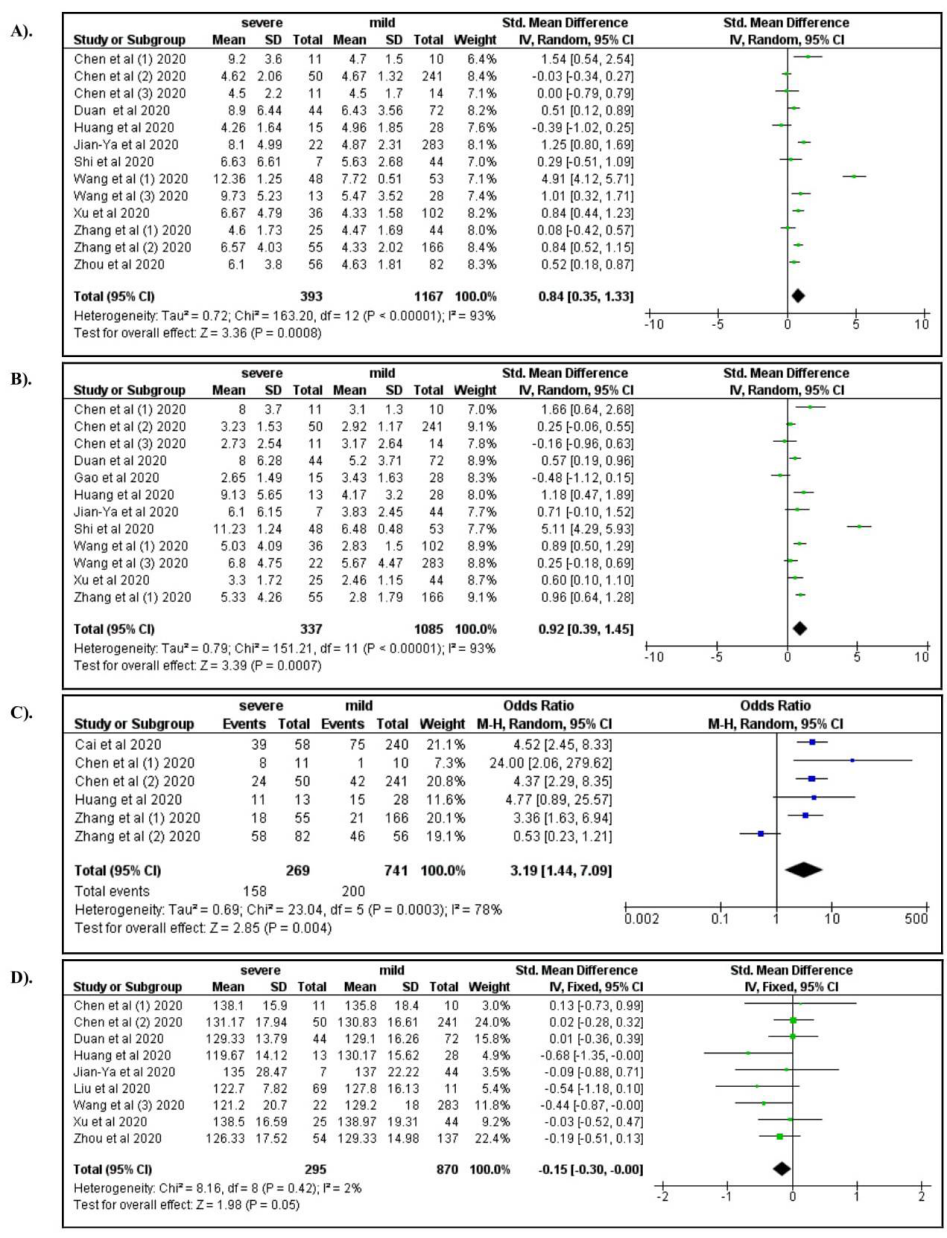
A forest of the association between complete blood count and the risk of severe COVID-19. **A**) White blood cells; **B**) Neutrophil count; **C**) Lymphocytopenia; **D**) Hemoglobin.

**Figure 6.**
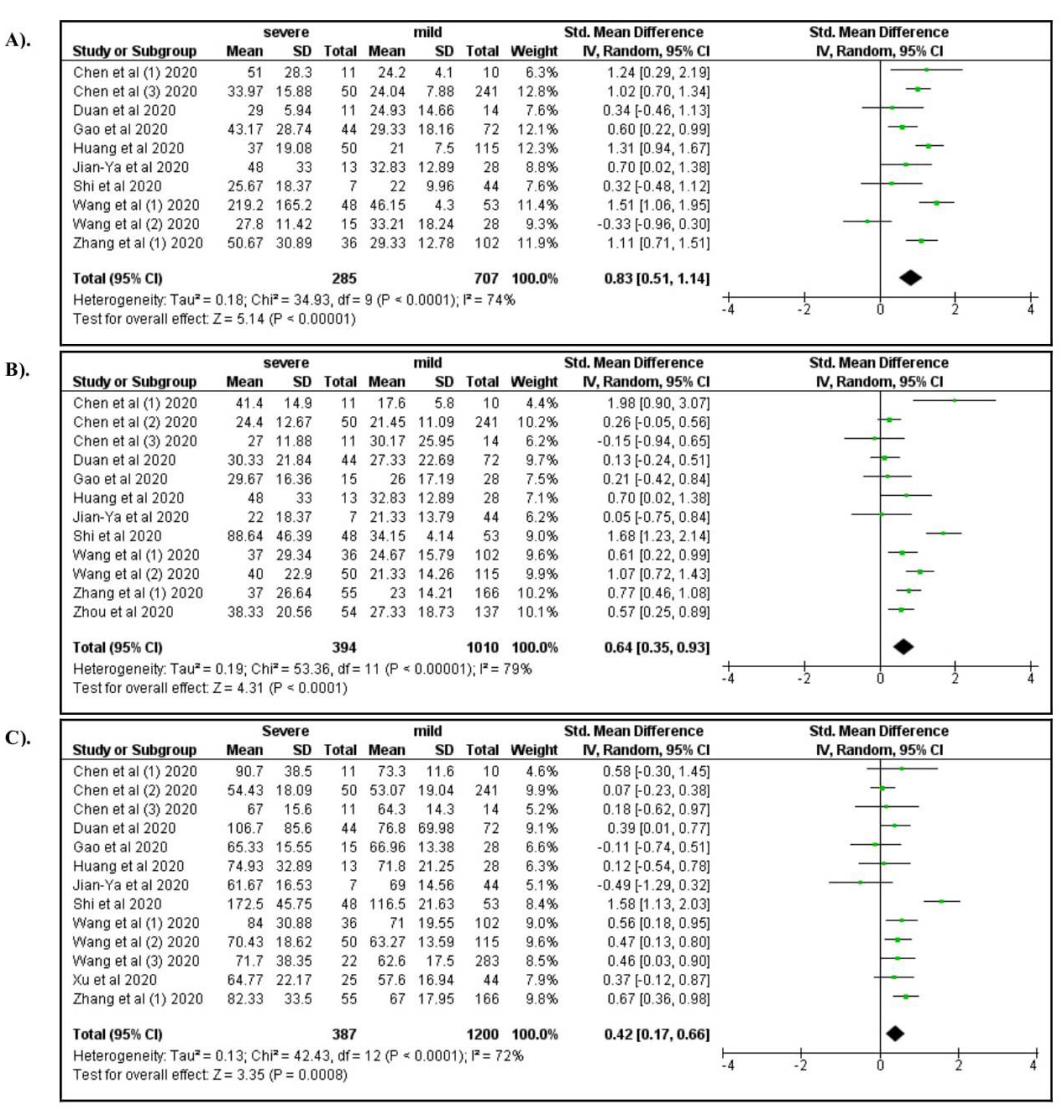
A forest plot of the association between the risk of severe COVID-19 and the levels of AST ( **A**), ALT ( **B**), and serum creatinine ( **C**).

**Figure 7.**
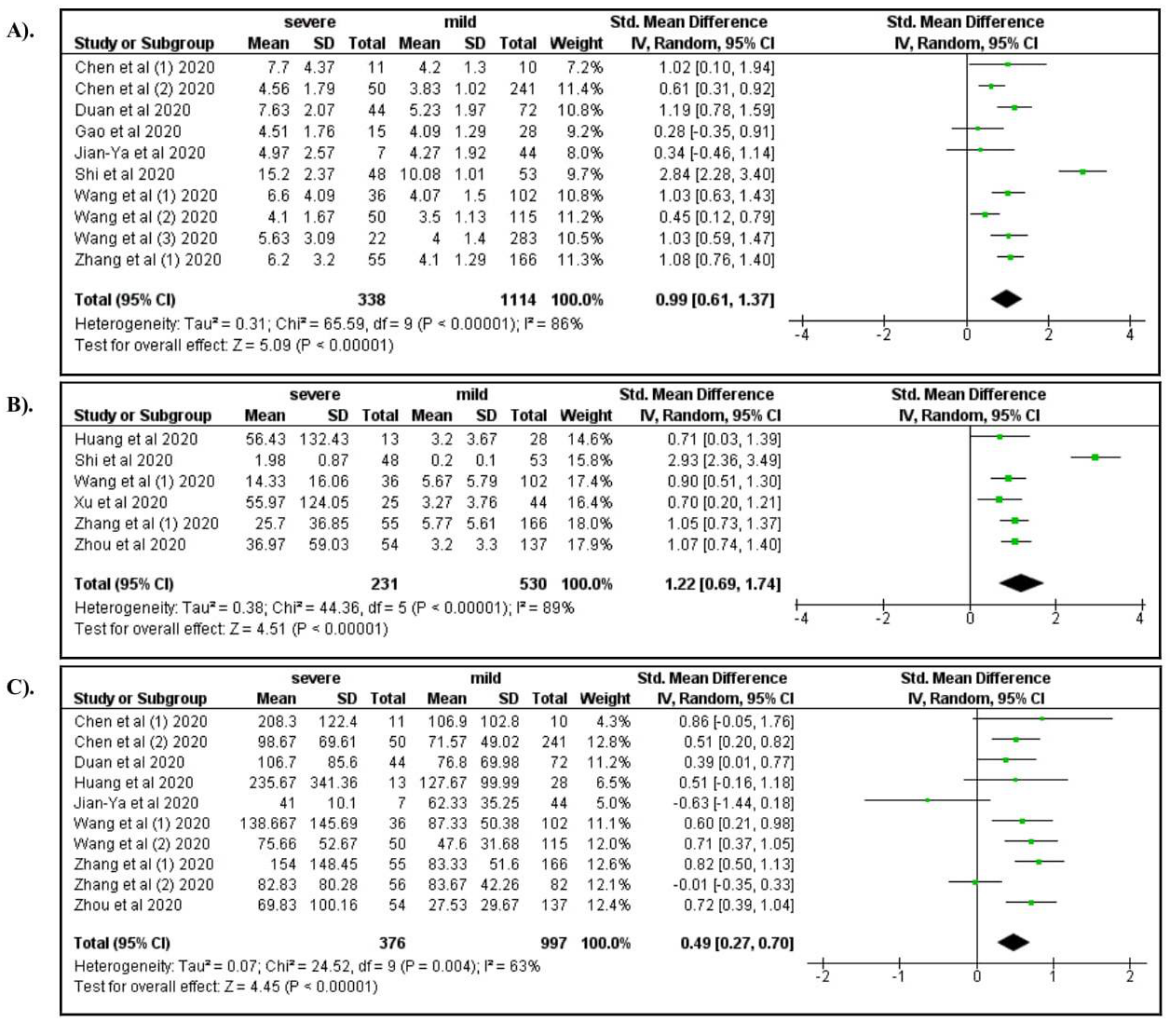
A forest plot of the association between the risk of severe COVID-19 and the levels of BUN ( **A**), Hs-troponin ( **B**), and creatine kinase ( **C**).

Among laboratory characteristics, severe COVID-19 was associated with elevated WBC count (OR: 4.92; 95% CI: 2.12, 11.31), increased neutrophil count (OR: 5.45; 95% CI: 2.04, 14.54), lymphocytopenia (OR: 3.19; 95% CI: 1.14, 7.07), and decreased hemoglobin levels (OR: 0.76; 95%CI: 0.58, 1.00) ( Figure 5A–D). Elevated levels of AST, ALT, and serum creatinine increased the risk for severe manifestations of COVID-19 (ORs 4.91, 3.23, and 2.14, respectively; Figure 6A–C). Elevated levels of BUN (OR: 6.15; 95% CI: 3.05, 12.37), Hs-troponin I (OR: 9.25; 95% CI: 3.51, 24.37), creatine kinase (OR: 2.44; 95% CI: 1.65, 3.62), Hs-CRP (OR: 14.27; 95% CI: 5.13, 39.71), IL-6 (OR: 6.68; 95% CI: 3.20, 13.94), D-dimer (OR: 6.19; 95% CI: 4.22, 9.08), ferritin (OR: 1.96; 95% CI: 1.06, 3.62), lactate dehydrogenase (OR: 8.28; 95% CI: 4.75, 14.46), procalcitonin (OR: 6.62; 95% CI: 3.32, 13.21), ESR (OR: 4.45; 95% CI: 2.56, 7.76), and CRP >8 (OR: 8.34; 95% CI: 1.85, 37.62) were also associated with severe COVID-19 ( Figure 7– Figure 9). A low risk of severe COVID-19 was associated with low leukocyte levels (OR: 0.59; 95% CI: 0.41, 0.87) and elevated lymphocyte levels (OR: 0.34; 95% CI: 0.23, 0.50).

**Figure 8.**
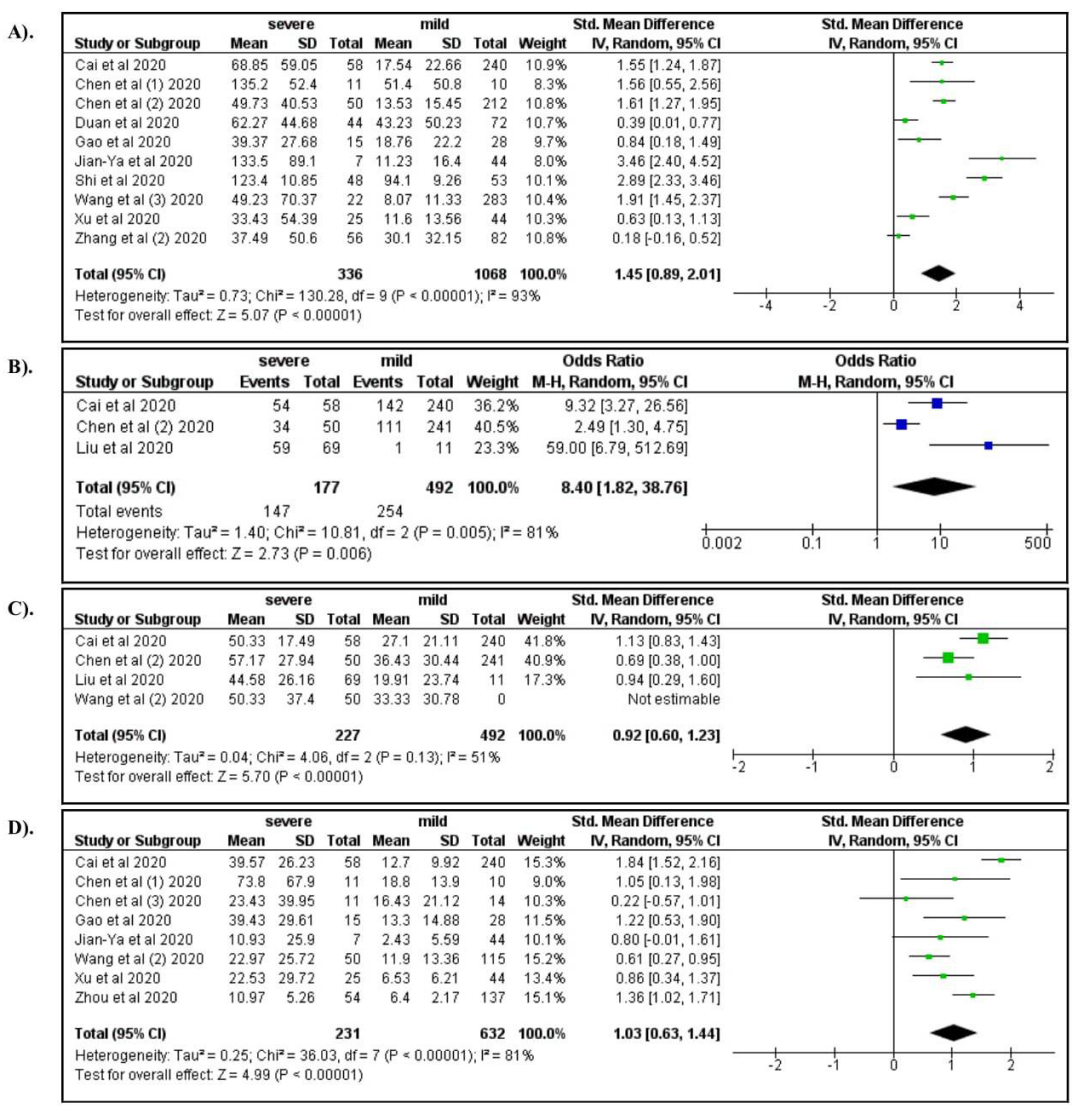
A forest plot of the association between the risk of severe COVID-19 and the levels of CRP ( **A**), Hs-CRP ( **B**), ESR ( **C**), and IL-6 ( **D**).

**Figure 9.**
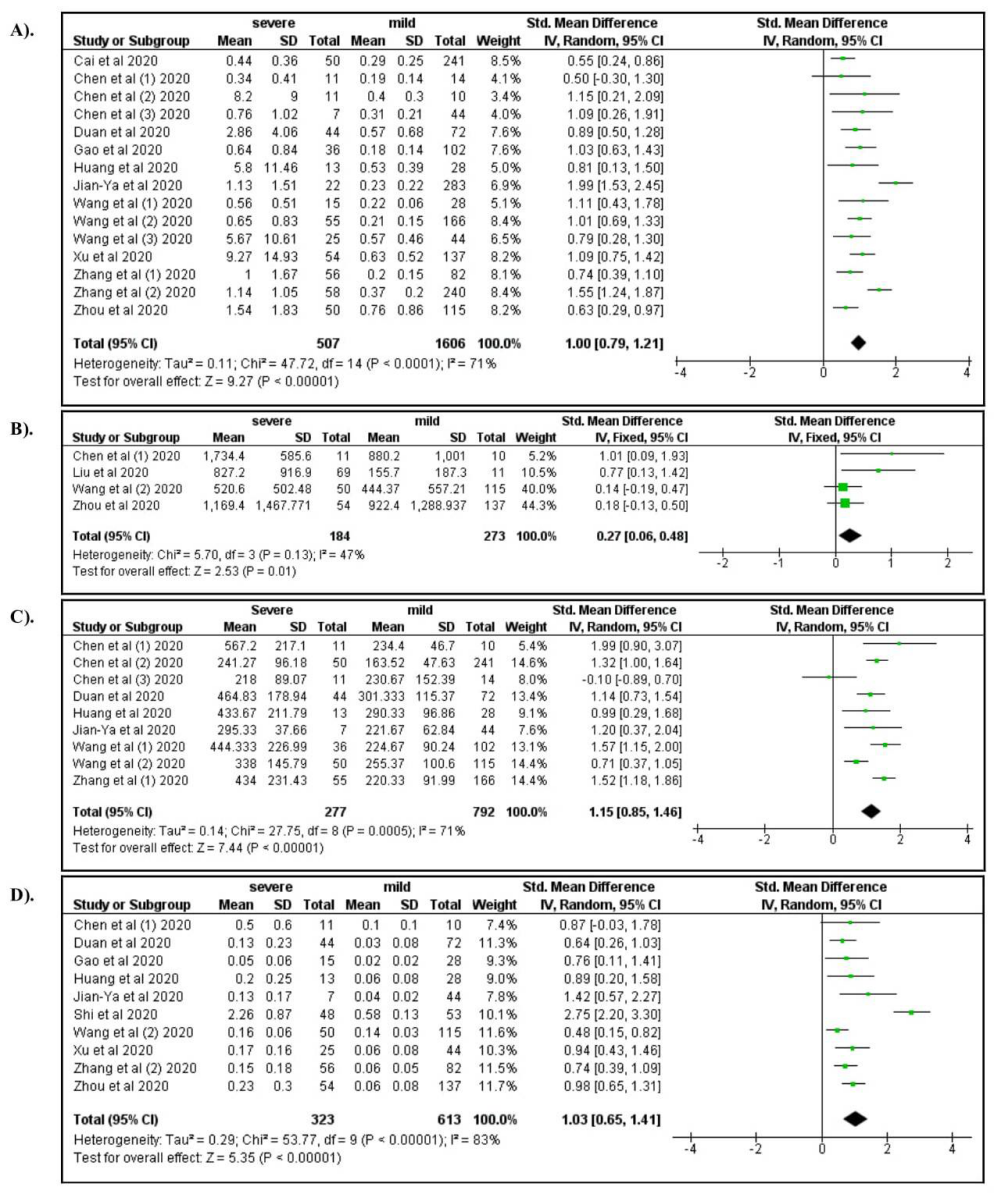
A forest plot of the association between the risk of severe COVID-19 and the levels of D-dimer ( **A**), serum ferritin ( **B**), lactate dehydrogenase ( **C**), and procalcitonin ( **D**).

### Source of heterogeneity

Heterogeneity was detected in the data of chronic kidney disease, cerebrovascular disease, cardiovascular disease, diabetes mellitus, hypertension, and malignancy among the comorbid factors analyzed. Therefore, we used the random effect model to analyze the data. The fixed effect model was used to analyze the data on chronic liver disease and chronic respiratory disease, as there was no evidence of heterogeneity. For clinical manifestations, the data on fever, cough, sore throat, dyspnea, diarrhea, anorexia, fatigue, temperature >38°C, respiratory rate, and diastolic blood pressure were analyzed using the random effect model while the rest of clinical manifestation data were analyzed using the fixed effect model.

Among laboratory parameters, evidence of heterogeneity was found in count of WBC, neutrophil, monocyte, lymphocyte, platelet, CD4, and CD8; the presence of lymphocytopenia and anemia; the levels of AST, ALT, total bilirubin, albumin, aPTT, PTT, serum creatinine, BUN, Hs-Troponin I, creatine kinase, IL-6, Hs-CRP, glucose, D-dimer, sodium, potassium, lactate dehydrogenase, and procalcitonin; elevated CRP; and ESR. Accordingly, the data were analyzed using the random effect model. The data for the remaining parameters were analyzed using the fixed effect model.

### Potential publication bias

We used Egger's test to assess the potential publication bias. Our cumulative calculation revealed that reporting or publication bias (p<0.05) existed with respect to chronic liver disease, expectoration, myalgia, abdominal pain, heart rate, leukocytosis, elevated ESR, and elevated IL-6 levels.

## Discussion

Our data suggest that comorbidities, such as chronic respiratory disease, cardiovascular disease, diabetes, and hypertension, were associated with a higher risk of severe COVID-19, among which, hypertension was the strongest risk factor. These results are consistent with those of previous meta-analyses ^43, 44^ that indicated that chronic respiratory disease, cardiovascular disease, diabetes, and hypertension are significantly associated with higher COVID-19 mortality. Hypertension and diabetes are also associated with higher mortality among patients with dengue fever, West Nile virus infection, Zika virus infection, and yellow fever ^45^. To date, no study has reported details of the primary mechanism underlying the association between severe COVID-19 and comorbid factors. However, immune responses might be the most crucial factor underlying this association. Patients with comorbidities such as cardiovascular disease, chronic respiratory disease, hypertension, and diabetes were observed to have a lower immunity status than healthy individuals ^46– 48^. Since COVID-19 primarily affects the respiratory tract ^49^, patients with chronic respiratory diseases might be at a higher risk of contracting severe COVID-19. In addition, endothelial dysfunction might also play a pivotal role ^50^.

COVID-19 is a novel disease, and the immune response of this disease is not completely understood. Our data suggest that elevated leukocyte and neutrophil levels and reduced lymphocyte levels are associated with severe COVID-19. In other viral infections, such as influenza, elevated leukocyte and neutrophil levels serve as important predictors of disease severity ^51^. The role of leukocytes in the pathogenesis of COVID-19 is conflicting. In most cases, viral infections have been observed to cause leukopenia ^52^. Furthermore, a study also reported that leukopenia was observed at a significantly higher frequency among COVID-19 patients than among non-COVID-19 patients ^53^. However, in our present study, we did not compare COVID-19 and non-COVID-19 patients. The major factor that seemed to affect our findings was the occurrence of cytokine storm in patients. In COVID-19, there is an immune system overreaction, which results in a cytokine storm. In this condition, leukocytes might be over-activated, which might lead to the release of high levels of cytokines ^54^. Consistent with our data, a study has confirmed that cytokine storm is significantly associated with severe COVID-19 ^55^. The theory underlying the role of neutrophils in COVID-19, as reported in our study, remains unclear. The speculations might be attributed to the involvement of neutrophil extracellular traps (NETs). While no study has assessed the precise role of NETs in COVID-19 pathogenesis, certain researchers speculate that SARS-CoV-2 might stimulate neutrophils to produce NETs, similar to several other viral pathogens ^56^. Furthermore, this might lead to neutrophil infiltration in pulmonary capillaries, organ damage, and the development of acute respiratory distress syndrome ^57^.

Low lymphocyte levels were observed in patients with severe COVID-19 compared with those with mild COVID-19. In the context of the immunological mechanism, our results might be contradictory. Lymphocyte subsets are known to play an important role in the action against bacterial, viral, fungal, and parasitic infections ^58^; therefore, the levels of circulating lymphocytes should increase. The immunological response in COVID-19 is unique and remains unclear. However, certain propositions might help describe our findings. First, coronaviruses infect human cells through ACE2 receptors ^59^. Since ACE2 receptors are also expressed by lymphocytes ^60^, the coronaviruses may enter lymphocytes and induce apoptosis. Second, the feedback mechanism between pro-inflammatory cytokines (such as IL-6) and lymphocytes might also explain our results. A study revealed that elevation in the levels of pro-inflammatory cytokines correlated with reduction in the levels of lymphocytes ^61^. Moreover, our findings also confirmed the significant elevation in the levels of IL-6. Third, ACE2 receptors are expressed by cells from various organs, including the thymus and spleen ^62^. As coronaviruses infect human cells through the ACE2 receptors, the spleen and thymus might also be damaged in patients with COVID-19, which would lead to lower levels of lymphocyte production. Fourth, lymphocyte proliferation requires a balanced metabolism, and metabolic disorders such as hyperlactic acidemia have been reported to disturb lymphocyte proliferation ^63^. Hyperlactic acidemia has been observed in patients with severe COVID-19 ^64^.

The studies included in this systematic review also suggest that the levels of D-dimer were significantly higher in patients with severe COVID-19. Coagulation in patients with COVID-19 has been a major concern, and the lack of reliable data and meta-analyses prevents a holistic comparison. Certain infectious diseases that cause abnormal coagulation have been associated with poor clinical outcomes ^65^. The theory behind this mechanism is not understood clearly. It is widely known that ACE2 receptors are important for the infection of host cells by SARS-CoV-2, and ACE2 receptors are expressed in various cells in the human body, including endothelial cells ^66^. Consequently, a massive inflammatory reaction may occur in endothelial cells owing to SARS-CoV-2 infection ^67^, which may lead to increased coagulation, disseminated intravascular coagulation ^68^, and increased fibrin degradation ^69^. High fibrin degradation leads to elevated levels of fibrinogen and D-dimer ^70^, which might also explain the occurrence of venous thromboembolism in critical patients of COVID-19 ^71^. In addition, a study with a short follow-up period also reported the existence of a dynamic correlation between the D-dimer levels and the severity of COVID-19 ^72^. Furthermore, pulmonary embolism and deep vein thrombosis were also observed in patients with severe COVID-19 ^73, 74^, which suggests that D-dimer might play a prominent role in governing the severity of COVID-19 patients.

We also observed that inflammatory markers, including elevated levels of CRP, ESR, and IL-6, were found both in patients with severe and mild COVID-19, with a significant increase detected in patients with severe COVID-19. Other variables associated with adverse outcomes, such as ferritin, lactate dehydrogenase, and procalcitonin levels, were found to be elevated predominantly in patients with severe COVID-19. Our findings were consistent with those of a previous meta-analysis ^75^, and indicated that high levels of CRP, lactate dehydrogenase, and ESR were associated with adverse outcomes in COVID-19. Another meta-analysis had also confirmed that elevated levels of IL-6 were observed in patients with COVID-19 who exhibited poor clinical outcomes ^76^. Therefore, the levels of CRP, ESR, IL-6, ferritin, procalcitonin, and lactate dehydrogenase can serve as potential markers for the evaluation of COVID-19 prognosis.

The high mortality rate and treatment failure in patients with COVID-19 can be attributed to the fact that COVID-19 affects multiple organs, including the lung, heart, kidney, and liver ^77^. Our data suggest that elevated levels of urea and creatinine, and not chronic kidney disease, were associated with severe COVID-19, which indicates that acute inflammation might be caused by SARS-CoV-2 infection. Previous meta-analyses have also reported findings consistent with our results ^78, 79^. Moreover, anatomical studies have reported significant renal inflammation in patients with severe COVID-19 ^75, 80, 81^. There might be two mechanisms by which SARS-CoV-2 induces renal inflammation. First, SARS-CoV-2 might directly infect renal tubular epithelial cells and podocytes through ACE2 receptors, which facilitates the targeted infection of certain cells by the virus. Consequently, acute tubular necrosis, podocytopathy, microangiopathy, and collapsing glomerulopathy might occur owing to the massive inflammation in renal tubular epithelial cells and podocytes ^82, 83^. Second, the binding between SARS-CoV-2 and ACE2 receptors might activate angiotensin II and induce cytokine production, which may lead to hypercoagulopathy and microangiopathy, and eventually cause renal hypoxia ^84, 85^.

Conversely, with respect to liver function, we observed that the levels of liver enzymes were higher in patients with severe COVID-19. Previous studies in this context have elucidated that ACE2 receptors are highly expressed in bile duct cells; therefore, infection of these cells by coronaviruses might lead to abnormalities in the levels of liver enzymes ^86^. However, a recent anatomical study on liver biopsy specimens from patients with severe COVID-19 revealed that moderate microvascular steatosis and mild lobular and portal activities were observed ^87^. These data suggest that it cannot be determined clearly whether the elevated levels of liver enzymes in patients with severe COVID-19 are caused by direct infection or by drug-induced liver injury. Therefore, further studies are required to elucidate the precise mechanism underlying the elevation of liver enzymes levels in patients with severe COVID-19.

Meta-analyses on this topic have been performed previously ^43, 44, 75, 76, 88– 91^. However, compared to previous studies, our study has the following strengths. The previous studies only reported limited factors, such as clinical manifestations ^43, 88, 90, 91^, laboratory findings ^76, 89^, or a combination of only clinical manifestations and laboratory findings ^75^. In our study, we included all comorbidities, clinical manifestations, and laboratory characteristics. Additionally, compared to previous studies, this study has a larger sample size; the data on 1,934 patients with mild and 1,644 patients with severe COVID-19 treated across 19 hospitals were retrieved. However, this study also has certain limitations. Certain crucial factors that might play an important role in the pathogenesis of COVID-19, including secondary infection, treatment, and immunological status were not controlled for. Our current findings should be interpreted with caution because the majority of studies included were cross-sectional, and the samples corresponding to the data analyzed originated only in China. Moreover, in our study, we did not perform the sub-group analysis according to the transmission area. As already reported, the transmission of COVID-19 in China was also affected by the transmission area ^92^. Therefore, this limitation might also affect the final findings of our study. Longitudinal studies may reveal more long-term impacts of SARS-CoV-2 infection ^93^.

## Conclusion

COVID-19 is an emergent infectious disease, and the major problem associated with it is the unknown pattern of disease development. We identified 34 factors that are associated with severe COVID-19. This might improve our understanding of COVID-19 progression and provide baseline data to compile or improve the prediction models for the estimation of COVID-19 prognosis. Moreover, our current findings may also contribute to guide the prioritization of high-risk target populations for vaccination.

## Data availability

### Underlying data

All data underlying the results are available as part of the article and no additional source data are required.

### Reporting guidelines

Figshare: PRISMA checklist for ‘Predictors of COVID-19 severity: a systematic review and meta-analysis’, https://doi.org/10.6084/m9.figshare.12813683.v1 ^94^

Data are available under the terms of the Creative Commons Attribution 4.0 International license (CC-BY 4.0).
