# Supplementary material for: Predictors of COVID-19 severity: a systematic review and meta-analysis
Source: F1000Res. 2021 Jan 6;9:1107. Originally published 2020 Sep 9. [Version 2] doi: 10.12688/f1000research.26186.2 (PMC7607482; doi:10.12688/f1000research.26186.2)
Supplement: Peer review history

# Peer review: Reviewer response for version 2

**DOI:** 10.5256/f1000research.30622.r76646

**Published:** 2021-01-19

**Authors:** Morteza Arab-Zozani

**Affiliations:** 1 Social Determinants of Health Research Center, Birjand University of Medical Sciences, Birjand, Iran

The authors responded to my comments clearly and the manuscript is accepted for publication in your journal.

Are the rationale for, and objectives of, the Systematic Review clearly stated?

Yes

Is the statistical analysis and its interpretation appropriate?

Yes

Are sufficient details of the methods and analysis provided to allow replication by others?

Partly

Are the conclusions drawn adequately supported by the results presented in the review?

Yes

Reviewer Expertise:

NA

I confirm that I have read this submission and believe that I have an appropriate level of expertise to confirm that it is of an acceptable scientific standard.

# Peer review: Reviewer response for version 1

**DOI:** 10.5256/f1000research.28897.r72568

**Published:** 2020-11-02

**Authors:** Annelies Wilder-Smith

**Affiliations:** 1 Heidelberg Institute of Global Health, University of Heidelberg, Heidelberg, Germany

The strength of this paper is the meta-analysis in terms of effect estimates. The weakness is the focus of data from China, while we should learn more from global data including the comparison between HIC and LMIC.

 In China, severity was also found to correlate with the force of infection, eg those in high transmission areas had more severe disease outcomes than those from lower transmission areas in China, see: Exposure to SARS-CoV-2 in a **hightransmission** setting increases the risk of severe **COVID**-19 compared with exposure to a low **transmission** setting?

 Chen D, Hu C, Su F, Song Q, Wang Z. **J Travel Med**. 2020 Aug 20;27(5):taaa094. doi: 10.1093/jtm/taaa094. ^1^

 The authors highlight the need for a scoring system for the prediction of severity. There is another reason why it is important to identify risk factors for severe disease: to guide prioritization of high risk target populations for vaccination

Are the rationale for, and objectives of, the Systematic Review clearly stated?

Yes

Is the statistical analysis and its interpretation appropriate?

Yes

Are sufficient details of the methods and analysis provided to allow replication by others?

Yes

Are the conclusions drawn adequately supported by the results presented in the review?

Yes

Reviewer Expertise:

COVID-19, Zika and dengue

I confirm that I have read this submission and believe that I have an appropriate level of expertise to confirm that it is of an acceptable scientific standard.

## References

[1] : Exposure to SARS-CoV-2 in a high transmission setting increases the risk of severe COVID-19 compared with exposure to a low transmission setting?. *Journal of Travel Medicine*.2020;27(5) : 10.1093/jtm/taaa094 10.1093/jtm/taaa094 PMC731386432502262

# Response

**Published:** 2020-11-03

**Authors:** Mudatsir Mudatsir

**Affiliations:** Universitas Syiah Kuala, Indonesia

Response to comments from reviewers:

 Reviewer 2#

 1. The strength of this paper is the meta-analysis in terms of effect estimates. The weakness is the focus of data from China, while we should learn more from global data including the comparison between HIC and LMIC. In China, severity was also found to correlate with the force of infection, eg those in high transmission areas had more severe disease outcomes than those from lower transmission areas in China, see: Exposure to SARS-CoV-2 in a high transmission setting increases the risk of severe COVID-19 compared with exposure to a low transmission setting? Chen D, Hu C, Su F, Song Q, Wang Z. J Travel Med. 2020 Aug 20;27(5):taaa094. doi: 10.1093/jtm/taaa094.1

 Response: The additional limitation has been added, as suggested

 2. The authors highlight the need for a scoring system for the prediction of severity. There is another reason why it is important to identify risk factors for severe disease: to guide prioritization of high risk target populations for vaccination.

 Response: The additional clinical implication has been added, as suggested.

# Peer review: Reviewer response for version 1

**DOI:** 10.5256/f1000research.28897.r71054

**Published:** 2020-09-21

**Authors:** Morteza Arab-Zozani

**Affiliations:** 1 Social Determinants of Health Research Center, Birjand University of Medical Sciences, Birjand, Iran

In this meta-analysis, you investigated the predictors of COVID-19 severity through the literature. You considered a topic of interest and provided a well-written manuscript. However, there are some things that will improve your reporting.

- Abstract, method section, please insert detail about critical/quality appraisal of the included studies.
- Abstract, method section, line 1, please remove " and extracted" from the text. It maybe causes a misunderstanding between this step and the data extraction step. 
- Method section, please remove line five. "the protocols for the ...". Mentioning the PRISMA is enough.
- Method section, eligibility criteria, (2) please mention the type of data for adequate data. what is adequate data?
- Method section, search strategy, why is Scopus not searched? You may have missed some articles that are only indexed in Scopus.
- Method section, search strategy, this sentence not related to this section. If you limit the search to EN publication then you need to change the verb. If not this sentence related to inclusion criteria. 
- Method section, search strategy, based on PRISMA, add at least one search strategy for one database as a supplement.
- Method section, data extraction, please added the country of origin for each study. The predictors may be different from one setting to another setting. 
- Method section, data extraction, please add details about how resolved disagreement between reviewers.
-  Method section, how did you handle the publication bias? 
- Result section, there is some problem in figure 1. Please fill it considering other related studies. The number for  "record screened" is incorrect.
- Result section, table 1, all studies are from China. If all studies are from China it is better to change the title. these are a predictor of severity in China. In my opinion, this is a limitation of your study.

Cheers

Are the rationale for, and objectives of, the Systematic Review clearly stated?

Yes

Is the statistical analysis and its interpretation appropriate?

Yes

Are sufficient details of the methods and analysis provided to allow replication by others?

Partly

Are the conclusions drawn adequately supported by the results presented in the review?

Yes

Reviewer Expertise:

Systematic review and meta-analysis in health and medical intervention

I confirm that I have read this submission and believe that I have an appropriate level of expertise to confirm that it is of an acceptable scientific standard.

# Response

**Published:** 2020-11-03

**Authors:** Mudatsir Mudatsir

**Affiliations:** Universitas Syiah Kuala, Indonesia

Response to comments from reviewers:

 Reviewer 1#

 1. In this meta-analysis, you investigated the predictors of COVID-19 severity through the literature. You considered a topic of interest and provided a well-written manuscript. However, there are some things that will improve your reporting. Abstract, method section, please insert detail about critical/quality appraisal of the included studies.

 Response: The description of the quality assessment of included papers has been added in the method of abstract.

 2. Abstract, method section, line 1, please remove " and extracted" from the text. It maybe causes a misunderstanding between this step and the data extraction step.

 Response: We have removed “and extracted”.

 3. Method section, please remove line five. "the protocols for the ...". Mentioning the PRISMA is enough.

 Response: PRISMA checklist may be interpreted as the general guideline in meta-analysis. The specific protocols may differ among meta-analysis studies; for example, the protocols of meta-analysis in gene polymorphism may differ from the protocols of meta-analysis in risk factors identification. In our manuscript, we referred to previous meta-analysis studies in the context of risk factors identification.

 4. Method section, eligibility criteria, (2) please mention the type of data for adequate data. what is adequate data?

 Response: The additional information related to adequate data has been provided.

 5. Method section, search strategy, why is Scopus not searched? You may have missed some articles that are only indexed in Scopus.

 Response: We also performed the searching strategy in Scopus as of 5 April 2020, however, we did not find additional articles. 

 6. Method section, search strategy, this sentence not related to this section. If you limit the search to EN publication then you need to change the verb. If not this sentence related to inclusion criteria. Response: English publication language has been added to eligibility criteria.

 7. Method section, search strategy, based on PRISMA, add at least one search strategy for one database as a supplement.

 Response: The additional database has been added as the additional database.

 8. Method section, data extraction, please added the country of origin for each study. The predictors may be different from one setting to another setting.

 Response: Country of origin has been added in data extraction.

 9. Method section, data extraction, please add details about how resolved disagreement between reviewers.

 Response: The additional sentence has been added to describe how to resolve the disagreement.

 10. Method section, how did you handle the publication bias?

 Response: The assessment of publication bias has been described in statistical analysis using Egger test. In the results, we presented in Tables 2 & 3.

 11. Result section, there is some problem in figure 1. Please fill it considering other related studies. The number for  "record screened" is incorrect.

 Response: In Figure 1, we used the template from PRISMA for the article selection pathway.

 12. Result section, table 1, all studies are from China. If all studies are from China it is better to change the title. these are a predictor of severity in China. In my opinion, this is a limitation of your study.

 Response: We tried to search articles in all regions, however, at the time frame of our searching, we only found articles in China.
